# Trends in incident diagnoses and drug prescriptions for anxiety and depression during the COVID-19 pandemic: an 18-month follow-up study based on the UK Biobank

**DOI:** 10.1038/s41398-023-02315-7

**Published:** 2023-01-19

**Authors:** Yue Wang, Fenfen Ge, Junren Wang, Huazhen Yang, Xin Han, Zhiye Ying, Yao Hu, Yajing Sun, Yuanyuan Qu, Thor Aspelund, Arna Hauksdóttir, Helga Zoega, Fang Fang, Unnur A. Valdimarsdóttir, Huan Song

**Affiliations:** 1 West China Biomedical Big Data Center, West China Hospital, Sichuan University, Chengdu, Sichuan China; 2 Med-X Center for Informatics, Sichuan University, Chengdu, Sichuan China; 3 Centre of Public Health Sciences, Faculty of Medicine, University of Iceland, Reykjavík, Iceland; 4 School of Population Health, Faculty of Medicine and Health, UNSW Sydney, Sydney, NSW Australia; 5 Institute of Environmental Medicine, Karolinska Institutet, Stockholm, Sweden; 6 Department of Epidemiology, Harvard T H Chan School of Public Health, Boston, MA USA

**Keywords:** Psychiatric disorders, Depression

## Abstract

Serious concerns have been raised about the negative effects of the COVID-19 pandemic on population psychological well-being. However, limited data exist on the long-term effects of the pandemic on incident psychiatric morbidities among individuals with varying exposure to the pandemic. Leveraging prospective data from the community-based UK Biobank cohort, we included 308,400 participants free of diagnosis of anxiety or depression, as well as 213,757 participants free of anxiolytics or antidepressants prescriptions, to explore the trends in incident diagnoses and drug prescriptions for anxiety and depression from 16 March 2020 to 31 August 2021, compared to the pre-pandemic period (i.e., 1 January 2017 to 31 December 2019) and across populations with different exposure statuses (i.e., not tested for COVID-19, tested negative and tested positive). The age- and sex-standardized incidence ratios (SIRs) were calculated by month which indicated an increase in incident diagnoses of anxiety or depression among individuals who were tested for COVID-19 (tested negative: SIR 3.05 [95% confidence interval 2.88–3.22]; tested positive: 2.03 [1.76–2.34]), especially during the first six months of the pandemic (i.e., March-September 2020). Similar increases were also observed for incident prescriptions of anxiolytics or antidepressants (tested negative: 1.56 [1.47–1.67]; tested positive: 1.41 [1.22–1.62]). In contrast, individuals not tested for COVID-19 had consistently lower incidence rates of both diagnoses of anxiety or depression (0.70 [0.67–0.72]) and prescriptions of respective psychotropic medications (0.70 [0.68–0.72]) during the pandemic period. These data suggest a distinct rise in health care needs for anxiety and depression among individuals tested for COVID-19, regardless of the test result, in contrast to a reduction in health care consumption for these disorders among individuals not tested for and, presumably, not directly exposed to the disease.

## Introduction

The coronavirus disease (COVID-19) pandemic continues to be a world-wide public health threat [1, 2]. Serious concerns have been raised about the negative effects of the pandemic on population psychological well-being, including new-onset psychiatric symptoms and disorders in people without preexisting problems [3, 4]. Indeed, a Canadian online survey reported that the proportion of generalized anxiety disorder and depression increased by 12% and 29%, respectively, during the pandemic [4]. Based on representative primary care data, Jacob et al. also found an increase in the number of patients newly diagnosed with anxiety disorder between March and June 2020 in Germany [5]. However, a significant reduction in first diagnoses of depression and anxiety between March and September 2020 was observed in UK, especially for adults of working age [6]. Likewise, psychiatric emergency consultations were also dramatically decreased across all psychiatric disorders during the lockdown (i.e., March-April 2020) in Paris and suburbs [7]. In terms of dispensing rate for antidepressants, a study in Canada showed a temporary reduction in April 2020 which then returned to the pre-pandemic levels by August 2020 [8].

Notably, previous studies have also suggested varying effects of the pandemic on population mental health across time [9] and degree of pandemic exposure [10–12]. For example, several countries have reported fluctuating levels of anxiety and depression across and between the different pandemic “waves” (i.e., according to numbers of COVID-19 cases, hospitalizations, and deaths) [13]. Based on cross-sectional data collected at the beginning of the pandemic, a study in China reported that clinical symptoms of anxiety and depression was predominately increased among patients with COVID-19 infection, compared to individuals under quarantine or the general public [11]. In the UK, Abel et al. observed an increased risk of fatigue, sleep problems, and psychotropic medication use among patients with negative COVID-19 test result, in contrast to matched controls in the general population [12]. Altogether, those findings indicated that patients who sought or received COVID-19 test may, irrespective of the test results, be at a higher exposure level of the pandemic (i.e., close to infected individuals or experiencing COVID-19 like symptoms) and thereby suffer a higher psychological burden.

Yet, limited data exist on the long-term effects of the pandemic on incident psychiatric disorders among individuals with varying exposure to the pandemic (e.g., COVID diagnosis), with careful control of potential confounding including seasonal effects. To this end, we leveraged routinely collected administrative data from the UK Biobank to assess trends in incident diagnoses of common psychiatric disorders – anxiety and depression – and prescriptions of respective psychotropic medications (i.e., anxiolytics and antidepressants) during the first 18 months of the pandemic, by history of COVID-19 testing (not tested, tested negative, and tested positive) as an indicator of pandemic exposure.

## Methods

### Study population and design

The UK Biobank is a population-based cohort study that recruited 502,507 men and women aged 40–69 years from England, Scotland, and Wales between 2006 and 2010, accounting for 5.5% of the UK population. Health-related outcomes of the participants are obtained regularly through linkage to multiple datasets [14]. Specifically, hospital inpatient data are obtained from Hospital Episode Statistics (HES) for England, Scottish Morbidity Record for Scotland, and Patient Episode Database for Wales. Mortality data are obtained from National Health Service (NHS) Digital and NHS Central Register. The hospital inpatient data (available since 1997) and mortality data (available since 2006) in England are currently updated to 30 September 2021. Furthermore, to facilitate COVID-19 related research, the UK Biobank also provided records of COVID-19 test results (by RT-PCR of nose/throat swab samples, available from 16 March 2020 to 30 September 2021) through linkage to Public Health England (PHE) [15], as well as updated primary care data obtained from two major general practice (GP) data system suppliers (EMIS and TPP) for approximately 450,000 UK Biobank participants in England (available from 1938 onward to 31 August 2021) [16]. More details about the UK Biobank are described elsewhere [17].

**Fig. 1:**
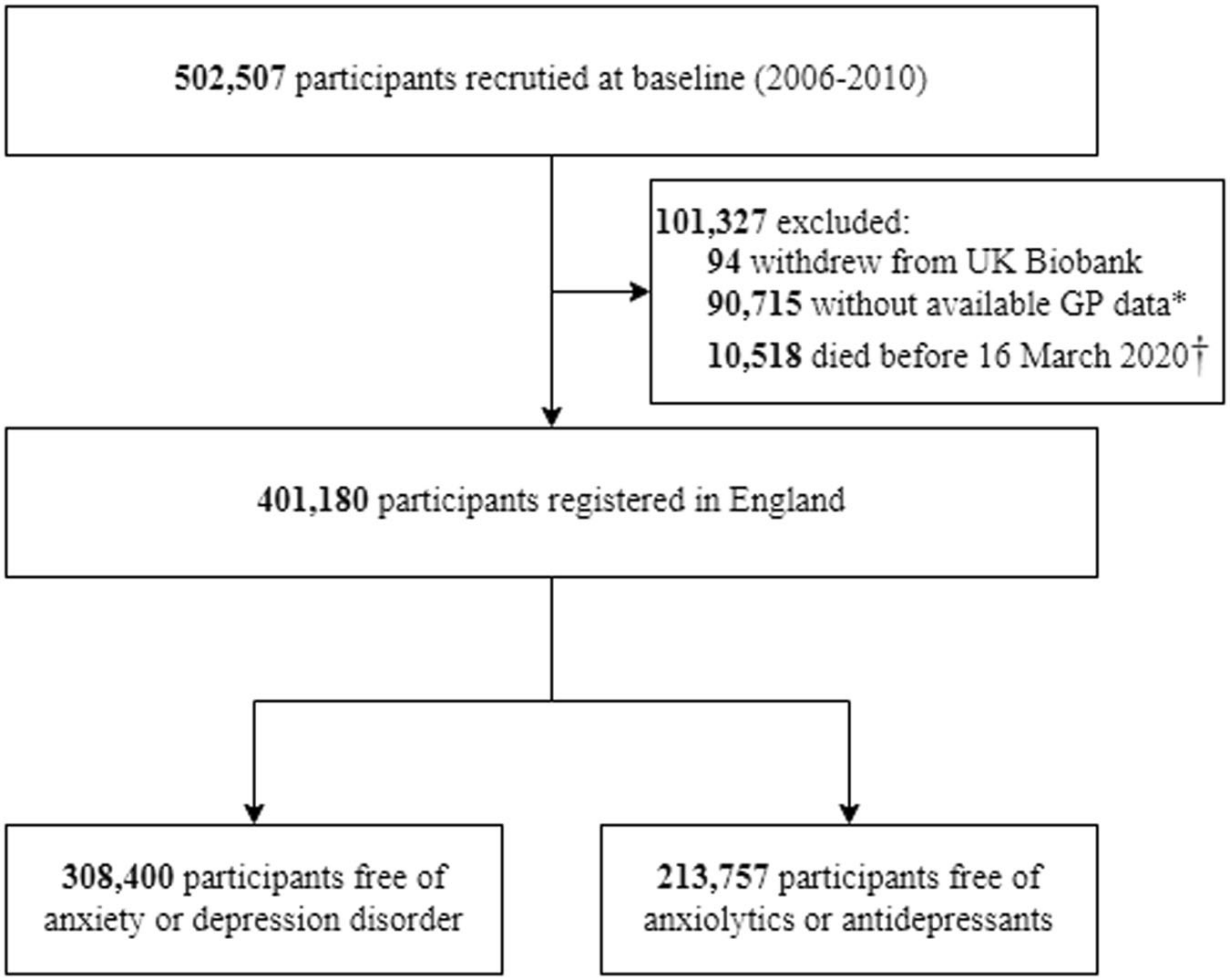
Flow chart of the COVID-19 pandemic exposure population. * The primary care data were only available for participants registered in England and registered at a practice using TPP or EMIS as their data system supplier. † Considering the results of COVID-19 tests was available in England through linkage to Public Health England database since 16 March 2020 onwards, thus, we defined the pandemic period start from 16 March 2020.

In this study, the outcomes of interest are incident diagnoses of anxiety and depression as well as prescriptions of the respective psychotropic medications during the COVID-19 pandemic. As the World Health Organization (WHO) declared the COVID-19 as a pandemic on 11 March 2020 and given the availability of COVID-19 related data in the UK Biobank, we defined the pandemic exposure period from 16 March 2020 to 31 August 2021. After excluding participants who withdrew from the UK Biobank (*n* = 94), had no available primary care data (*n* = 90,715), or died before 16 March 2020 (*n* = 10,518), we included 401,180 participants registered in England in the study (Fig. 1). Among these participants, we first constructed a cohort for the analysis of incident diagnoses of depression or anxiety, including 308,400 participants who were free of anxiety (International Classification of Diseases, Tenth Revision (ICD-10), F40-F41) and depression (ICD-10, F32-F33), as of 16 March 2020, to examine the trend of incident diagnoses of these psychiatric disorders during the COVID-19 pandemic. Then, to study the trend of incident prescriptions of respective psychotropic medications (i.e., anxiolytics and antidepressants), we constructed another cohort including 213,757 participants who were free of prescription of these medications on 16 March 2020. We followed up those participants from baseline (i.e., 16 March 2020) until the occurrence of any outcomes of interest, death, or the end of follow­up (i.e., 31 August 2021). In comparison, we used the three preceding years before the pandemic (i.e., from 1 January 2017 to 31 December 2019) as the reference pre-pandemic period. Supplementary Fig. S1 shows the flow chart of the pre-pandemic population selection.

The UK Biobank collected all data after written informed consent obtained from each participant and the study has full ethical approval from the NHS National Research Ethics Service (16/NW/0274). This present study was also approved by the biomedical research ethics committee of West China Hospital (2020.661).

### Ascertainment of anxiety, depression, and psychotropic medications

Diagnoses of anxiety and depression were retrieved from the UK Biobank hospital inpatient data (according to ICD-10 codes) [18] and primary care data (according to SNOMED CT, local EMIS codes, Clinical Terms Version 3, and Local TPP codes) [19]. The diagnoses of psychiatric disorders from HES in England have been validated, indicating a positive predictive value of up to 75% for depression and <60% for anxiety [20]. Prescription of psychotropic medications was ascertained through mapping the UK Biobank prescription codes (i.e., dm+d and local EMIS codes) to their corresponding active ingredients using dm+d XML Transformation Tool and UK Biobank Data-Coding 7678 resource [19]. The active ingredients were then matched to the WHO Atomical Therapeutic Chemical (ATC) classification system [21], and the ingredients (e.g., doxepin, esketamine) with more than one matched ATC codes were discarded as it is difficult to disentangle therapeutic targets. We extracted information (i.e., prescription code and date) about anxiolytics (N05B) and antidepressants (N06A) in the analysis. Supplementary Table S1 provides a list of the active ingredients and their corresponding ATC codes used in this study.

### Stratification by history of COVID-19 testing

Accounting for the reported variations in psychological implications of COVID-19 pandemic among populations with different exposure level, we categorized the participants into three pandemic exposure groups, using the history COVID-19 testig as an indicator (i.e., not tested, tested negative, and tested positive). As the testing status could change over time, the categorization was done in a time-varying manner, namely that all participants were in the “not tested” group on 16 March 2020, while those with a record of negative test result in PHE were moved to the “tested negative” group on the date of the corresponding COVID-19 test. Further, individuals not tested or tested negative could move to “tested positive” group on the date of a positive test result as recorded in PHE, or the date of a COVID-19 diagnosis (ICD-10 codes, U07.1 and U07.2) as documented in the UK Biobank inpatient hospital data, if any. Although the UK Biobank population is not representative of the entire UK population, we found largely similar trend in the daily number of COVID-19 cases in the study cohort as that in the whole England (i.e., publicly announced number of cases, Supplementary Fig. S2) [22].

### Statistical analysis

We first calculated the crude incidence rates (IRs, dividing the number of outcomes by accumulated person-years at risk) of the incident diagnoses of anxiety or depression as well as prescriptions of psychotropic medications during the pandemic period, respectively. We then calculated the IRs by history of COVID-19 testing (i.e., not tested, tested negative, and tested positive). The comparison between the pandemic and pre-pandemic periods was achieved by applying indirect standardization method to calculate the standardized incidence ratios (SIRs, standardized by age and sex) and their 95% confidence intervals (95%CIs). As psychological well-being may be influenced by seasonal variation, we further calculated the monthly SIRs during the 18-month pandemic period, using the average month-, sex-, and age-specific incidence rate of the studied outcomes from the pre-pandemic period as reference.

Moreover, we performed separate analyses for anxiety and depression as well as for anxiolytics and antidepressants. In addition, to test the robustness of our results to severe somatic conditions, we repeated all analyses after excluding participants with severe somatic diseases (i.e., those included in Charlson comorbidity index calculation, see Supplementary Table S2) on 16 March 2020. We also conducted a sensitivity analysis by re-defining the pandemic period as 18 May 2020 (i.e., when COVID-19 test was available to everyone with symptoms in the UK) [23] to 31 August 2021.

## Results

Among the 308,400 participants of the cohort for incident diagnoses of anxiety or depression (mean age = 68.2, 47.9% male) (Table 1), 4 439 received their first diagnosis of anxiety or depression during the pandemic period, corresponding to an IR of 9.7 per 1000 person-years. The IR was 7.5, 33.2, and 21.3 per 1000 person-years for participants who had not been tested, had tested negative, and tested positive for COVID-19, respectively. When compared with the IR of the pre-pandemic period (10.4 per 1000 person-years), we observed a decrease in incident diagnoses of anxiety or depression among participants not tested for COVID-19 (SIR 0.70 [95% CI 0.67–0.72]), but an increase in incident diagnoses of anxiety or depression among participants tested for COVID-19, irrespective of test result (tested negative: 3.05 [2.88–3.22]; tested positive: 2.03 [1.76–2.34]). Among the 213 757 participants included in the cohort of incident prescriptions of psychotropic medications (mean age = 67.9, 52.3% male) (Table 1), we observed largely similar risk patterns. For instance, there was a decrease in incident medication prescriptions among those not tested (0.70 [0.68–0.72]), but an increase in incident medication prescriptions among those tested for COVID-19 (tested negative: 1.56 [1.47–1.67]; tested positive: 1.41 [1.22–1.62]) during the pandemic period, compared with the pre-pandemic period.

**Table 1:** Characteristics of the pandemic cohort.

	Incident diagnoses of psychiatric disorder analysis		Incident prescriptions of psychotropic medication analysis	
	Tested for COVID-19 (*n* = 86,529)	Total (*n* = 308,400)	Tested for COVID-19 (*n* = 56,025)	Total (*n* = 213,757)
Mean Age at study period	68.6 (8.2)	68.2 (8.1)	68.2 (8.3)	67.9 (8.1)
Age at study period				
Lower than median (<69 years)	38,652 (44.7%)	143,434 (46.5%)	26,095 (46.6%)	102,662 (48.0%)
Higher than median (≥69 years)	47,877 (55.3%)	164,966 (53.5%)	29,930 (53.4%)	111,095 (52.0%)
Sex				
Female	43,407 (50.2%)	160,555 (52.1%)	25,248 (45.1%)	102,014 (47.7%)
Male	43,122 (49.8%)	147,845 (47.9%)	30,777 (54.9%)	111,743 (52.3%)
Townsend deprivation index				
Lower than median (<−2.15)	43,348 (50.1%)	157,725 (51.1%)	28,266 (50.5%)	110,227 (51.6%)
Higher than median (≥−2.15)	43,069 (49.8%)	150,315 (48.7%)	27,690 (49.4%)	103,262 (48.3%)
Unknown	112 (0.1%)	360 (0.1%)	69 (0.1%)	268 (0.1%)
Ethnicity				
White	80,465 (93.0%)	287,622 (93.3%)	52,204 (93.2%)	199,806 (93.5%)
Mixed	548 (0.6%)	1860 (0.6%)	368 (0.7%)	1280 (0.6%)
Black	2304 (2.7%)	8203 (2.7%)	1407 (2.5%)	5455 (2.6%)
Asian	1834 (2.1%)	5974 (1.9%)	1156 (2.1%)	3996 (1.9%)
Other	935 (1.1%)	3042 (1.0%)	592 (1.1%)	2045 (1.0%)
Unknown	452 (0.5%)	1699 (0.6%)	298 (0.5%)	1175 (0.5%)
Annual Income				
≤£ 18,000	14,624 (16.9%)	51,482 (16.7%)	8408 (15.0%)	32,427 (15.2%)
£ 18,000–£ 30,999	17,945 (20.7%)	65,657 (21.3%)	11,330 (20.2%)	44,882 (21.0%)
£ 31,000–£ 51,999	19,402 (22.4%)	70,671 (22.9%)	13,062 (23.3%)	50,497 (23.6%)
£ 52,000–£ 100,000	16,131 (18.6%)	57,775 (18.7%)	11,506 (20.5%)	43,253 (20.2%)
≥£ 100,000	5378 (6.2%)	16,337 (5.3%)	4085 (7.3%)	12,838 (6.0%)
Unknown	13,049 (15.1%)	46,478 (15.1%)	7634 (13.6%)	29,860 (14.0%)
College education				
Without	42,749 (49.4%)	152,771 (49.5%)	27,123 (48.4%)	103,999 (48.7%)
With	28,081 (32.5%)	102,532 (33.2%)	20,346 (36.3%)	77,723 (36.4%)
Unknown	15,699 (18.1%)	53,097 (17.2%)	8556 (15.3%)	32,035 (15.0%)
Body mass index, kg/m^2^^a^				
<18.5	318 (0.4%)	1379 (0.4%)	206 (0.4%)	1007 (0.5%)
18.5–24.9	26,402 (30.5%)	101,551 (32.9%)	17,858 (31.9%)	73,030 (34.2%)
25–29.9	37,425 (43.3%)	133,261 (43.2%)	24,543 (43.8%)	93,138 (43.6%)
≥30	21,866 (25.3%)	70,586 (22.9%)	13,087 (23.4%)	45,538 (21.3%)
Unknown	518 (0.6%)	1623 (0.5%)	331 (0.6%)	1044 (0.5%)
Charlson comorbidity index^b^				
0	48,638 (56.2%)	205,458 (66.6%)	33,683 (60.1%)	149,850 (70.1%)
≥1	37,891 (43.8%)	102,942 (33.4%)	22,342 (39.9%)	63,907 (29.9%)

^a^The Body mass index was calculated using weight kilograms (kg) by the square of height in meters (m^2^).

^b^The Charlson comorbidity index was calculated based on the UK Biobank inpatient hospital data.

**Fig. 2:**
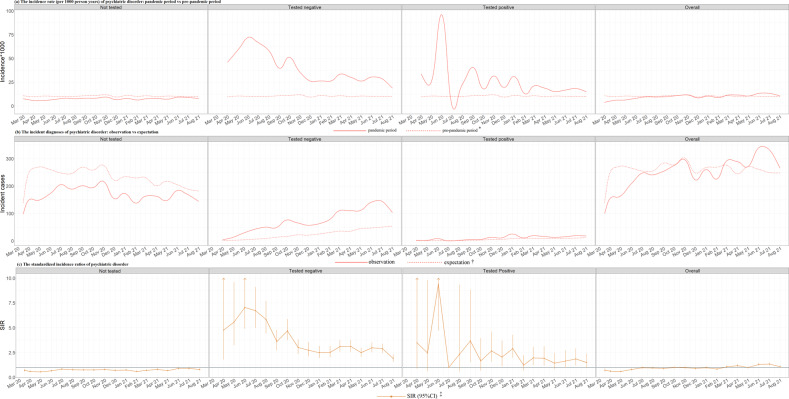
Period specific incident diagnoses of anxiety and depression disorder. Note, IR incidence rate, SIR standardized incidence ratio. * Three preceding years as the pre-pandemic period (i.e., from 1 January 2017 to 31 December 2019). † The expectation was calculated by multiplying the number of persons by the average month-specific, sex-specific, and age-specific (1-year strata) incidence rate derived from pre-pandemic period. ‡ The standardized incidence ratio was calculated by comparing the number of observed incident cases with the expectation.

**Fig. 3:**
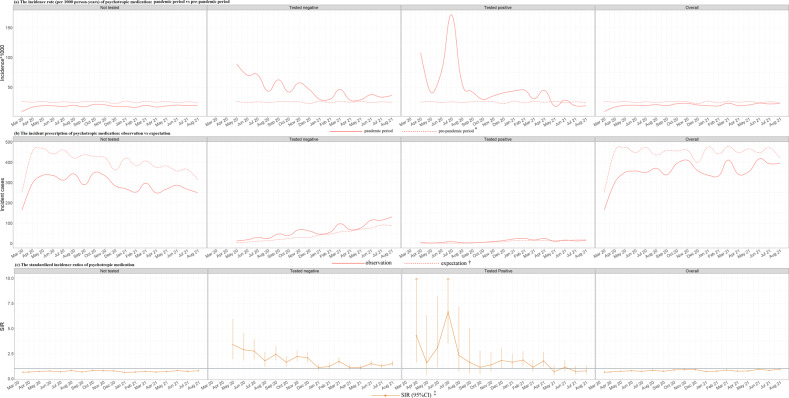
Period specific incident prescription of anxiolytics and antidepressants. Note, IR incidence rate, SIR standardized incidence ratio. * Three preceding years as the pre-pandemic period (i.e., from 1 January 2017 to 31 December 2019). † The expectation was calculated by multiplying the number of persons by the average month-specific, sex-specific, and age-specific (1-year strata) incidence rate derived from pre-pandemic period. ‡ The standardized incidence ratio was calculated by comparing the number of observed incident cases with the expectation.

The analysis of seasonal variation showed a lower overall risk for incident diagnoses of anxiety or depression during the pandemic period when compared to the pre-pandemic period, except in June 2021 (1.30 [1.17–1.45]) and July 2021 (1.34 [1.20–1.49]) (Fig. 2). We again obtained different results when separately analyzing by history of COVID-19 testing, with an increased incidence rate among individuals tested for COVID-19, especially during the beginning of the pandemic (i.e., March 2020 - September 2020; tested negative: SIRs 3.61–7.05, tested positive: SIRs 2.33–9.38), whereas a decreased incidence rate among those not tested during the entire 18-month period (SIRs 0.55–0.90). Similar results were obtained for incident prescriptions of anxiolytics or antidepressants (Fig. 3). There was an increase in incidence rate of these psychotropic medications during the first six months of the pandemic among individuals tested for COVID-19 (tested negative: SIRs 1.78–3.38; tested positive: SIRs 1.57–6.66), but a constantly lower incidence rates among those not tested for COVID-19 (SIRs 0.64–0.82).

We observed largely comparable results when analyzing anxiety and depression, as well as anxiolytics and antidepressants, separately (Supplementary Figs. S3 and S4). These findings remained robust after excluding participants with pre-existing severe somatic diseases (Supplementary Figs. S5 and S6) and after re-defining the pandemic period from 18 May 2020 (Supplementary Figs. S7 and S8).

## Discussion

In general, the findings of this study based on the UK Biobank indicate that individuals tested for COVID-19, irrespective of the test result, experienced a rise in the incident diagnosis of anxiety and depression as well as respective medication use, particularly at the beginning of the pandemic. In contrast, we observed a decrease in incident diagnoses of anxiety and depression as well as prescriptions of respective psychotropic medications during the pandemic in the general population not tested for COVID-19, yet with an increase beyond pre-pandemic levels in mid 2021.

The finding on lower incidence rates of anxiety and depression as well as prescriptions of psychotropic medications in the general population may reflect a decrease in general psychiatric healthcare utilization during the first 18 months of the pandemic. In line with our findings, a drop in psychiatric emergency visits and hospital admission during the pandemic has previously been reported in other European countries [24, 25]. For instance, in Italy, a 37.5% decrease in the number of psychiatric emergency consultations was observed during the lockdown (March-May 2020) and 17.9% after the lockdown period [26]. Similarly, a retrospective study in Spain suggested that the most marked reduction in admission rates during lockdown (March-June 2020) was observed for anxiety-related disorders [27]. Moreover, addition to the political restriction [23], as SARS-CoV-2 is highly infectious, people may intentionally delay or avoid seeking medical care during the unprecedented period. The findings of Czeisler et al. reported that 12% of US adults delayed or avoided emergency care and 32% avoided routine medical care [28]. Existing evidence about the trends of incident antidepressants and anxiolythics prescriptions during the pandemic is inconsistent. For instance, Antonazzo et al suggested an abrupt reduction of incident antidepressants use during the lockdown in Italy, with a following rebound during the post-lockdown period [29], while a continuing increase in new antidepressant use was observed during the first year of the pandemic in Australia [30]. Furthermore, age-group differences have been noted in the consumption of psychotropic medications, with the most significant increase found in the younger population [30, 31].

The observed decrease in incident diagnoses in the general population appeared to be mainly attributable to individuals not tested for COVID-19. Thus, it is also possible that the decrease in incident diagnoses might reflect lower prevalence of psychiatric morbidity in this population because people might have spent more time with family and friends or had slower pace of daily life during the pandemic [32]. Indeed, the protective effects of family and friends’ support on depressive symptoms and PTSD symptoms are well-documented [33, 34]. Additionally, a recent study from France reported improved sleep in the general population during the pandemic which might positively affect mental health [35]. Regardless, as the effect of pandemic on population mental health is complex and profound, future research focused on both negative and positive aspects of the crisis is needed.

In contrast, the increase in incident psychiatric morbidities among individuals tested for COVID-19 may have several explanations. It is conceivable that people tested for COVID-19 might have been exposed to infected individuals or be symptomatic, and the psychological burden of suspecting to be infected may contribute to the observed increase. Testing for COVID-19 itself and waiting for the test result might also be stressful and associated with increased anxiety [36]. One study showed that more than half of patients suspected to have COVID-19 had increased psychotropic medication prescriptions [37]. Alternatively, individuals who had taken a COVID-19 test might also have temporarily reduced social contact, i.e., being quarantined or isolated, to avoid infecting others, resulting in negative impact on mental health [38]. On the other hand, studies conducted by our [39, 40] and other research groups [41] demonstrated a bidirectional association between COVID-19 and psychiatric disorders, suggesting that individuals with propensity for psychiatric disorders may be more vulnerable for COVID-19 infection and adverse outcomes. With respect to the test result, we observed a similar increased risk of anxiety and depression for individuals with both negative and positive results, which is in agreement with Abel et al.’s finding [12]. Likewise, when compared to tested negative individuals, a Danish registry study found that there was no significant increase in risk for initiation of new antidepressants among tested positive individuals [42]. However, Klaser et al reported that symptoms of anxiety and depression were slightly more prevalent among individuals tested positive compared to those tested negative for COVID-19 [43].

The most notable increase in incident diagnoses of anxiety and depression among COVID-19 tested group was found at the beginning of the pandemic. One reason for the temporary spike at the initial stage of the pandemic is the uncertainty about the virus at that timpoint [44]. Fear of being infected or infecting others may trigger psychological distress. The less or insignificantly elevated incidence rates in later periods of pandemic in this population may be attributed to population adaptation of the COVID-19 crisis [45] and the wide implementation of vaccination in the UK [46], which might have reduced stress or distress. Regardless, as the increased incidence rates persisted nearly the entire 18-month pandemic period, further research with longer follow-up is warranted to understand the continued impact of the pandemic on population mental health.

Study strengths include the use of timely updated UK Biobank primary care and PHE data, which enabled us to explore the long-term psychological consequences among individuals with different history of COVID-19 testing during the pandemic. Moreover, the application of self-comparison analysis, where the rate of incident events during the pandemic period was compared with the corresponding age- and sex-specific rate of the three preceding years in the same study population, inherently allows for the control for potential confounders that are constant over time, including genetic factors and many environmental factors that did not change greatly within the study period. In addition, we carefully considered the impact of seasonal variation through analyzing monthly SIRs.

One major concern of our study is the varying access to COVID-19 testing between groups of the study participants and during different parts of the pandemic period. We therefore performed sensitivity analyses through excluding participants with severe pre-pandemic comorbidities or re-defining pandemic period from 18 May 2020, which suggested some but limited influence of this concern. Also, regardless of test result, it is further possible that some other factors determined the likelihood of taking a COVID-19 test. For example, seeking or receiving a COVID-19 tests may indicate higher level of medical care utilization and therefore increased possibility of being diagnosed or prescribed for existing psychiatric symptoms (i.e., surveillance bias). However, the impact of such a concern, if any, should be constant throughout the whole study period, whilst the change in incident diagnoses and drug prescriptions for anxiety and depression was indeed time-varying, suggesting potential contribution of other causes, such as temporary psychological burden caused by COVID-19-related exposures or reduced social contacts. In addition, considering the overloaded healthcare system during the pandemic period, our outcome identification strategy (using diagnoses and drug prescriptions from both primary care and hospital inpatient data) is likely to only capture patients with more severe psychiatric symptoms. Conditions for individuals with less severe symptoms who did not seek medical care need to be studied in future studies. Likewise, as our identification of COVID-19 cases relies solely on RT-PCR testing (but not self-tests which became available in UK since January 2021 [47]), individuals testing positive for COVID-19 on RT-PCR are more likely represented by those have been symptomatic. Moreover, the sample size of individuals tested positive for COVID-19 was relatively small yielding insufficient precision to depict the monthly variations during the pandemic period, and the absence of data on acute COVID-19 severity prohibit the assessment of trends by varying COVID-19 severity. Also, varying mortality rates in the studied groups and the resulting competing risks may have resulted in an underestimated relative risks of psychiatric outcomes, particularly among individuals with COVID-19 [48]. Furthermore, the UK Biobank primary care data are only available for participants registered at TPP and EMIS system in England, whereas the prescriptions of psychotropic medications cannot be assumed to equal actual consumption of these medications [19]. Finally, the UK Biobank only recruited 5.5% of the UK population, mainly elderly white people, thus, our results cannot necessarily be generalized to the entire UK population or other populations.

## Conclusion

In conclusion, the findings of this study suggest differential population mental health care utilization during the COVID-19 pandemic by level of pandemic exposure indicated by history of COVID-19 testing. Compared to the pre-pandemic period, we observed a decrease in incident diagnoses of anxiety and depression as well as prescriptions of respective psychotropic medications among individuals not tested for COVID-19, but an increase in incident psychiatric morbidity among those tested for COVID-19, irrespective of the test result, particularly at the beginning of COVID-19 pandemic.

## Supplementary information

Supplementary

## Data Availability

Data from the UK Biobank (http://www.ukbiobank.ac.uk/) are available to all researchers upon making an application.

## Code availability

All analyses were performed in R version 4.0. Codes associated with the current submission is available and can be requested by contacting the corresponding author.
