# Supplementary material for: Trends in incident diagnoses and drug prescriptions for anxiety and depression during the COVID-19 pandemic: an 18-month follow-up study based on the UK Biobank
Source: Transl Psychiatry. 2023 Jan 19;13:12. doi: 10.1038/s41398-023-02315-7 (PMC9849101; doi:10.1038/s41398-023-02315-7)
Supplement: Supplementary file 1 — Supplementary [file 41398_2023_2315_MOESM1_ESM.docx]

Supplementary

**Supplementary Tables**

Table S1 Active ingredients and Anatomical Therapeutic Chemical (ATC) code of studied psychotropic medications

| **ATC code** | **Active ingredients** | **ATC code** | **Active ingredients** | **ATC code** | **Active ingredients** |
| --- | --- | --- | --- | --- | --- |
| N05BA01 | Diazepam | N06AA07 | Lofepramine | N06AX01 | Oxitriptan |
| N05BA02 | Chlordiazepoxide | N06AA09 | Amitriptyline | N06AX02 | Tryptophan |
| N05BA04 | Oxazepam | N06AA10 | Nortriptyline | N06AX03 | Mianserin |
| N05BA06 | Lorazepam | N06AA11 | Protriptyline | N06AX05 | Trazodone |
| N05BA08 | Bromazepam | N06AA16 | Dosulepin | N06AX06 | Nefazodone |
| N05BA09 | Clobazam | N06AA17 | Amoxapine | N06AX09 | Viloxazine |
| N05BA11 | Prazepam | N06AA21 | Maprotiline | N06AX11 | Mirtazapine |
| N05BA12 | Alprazolam | N06AB03 | Fluoxetine | N06AX12 | Bupropion |
| N05BB01 | Hydroxyzine | N06AB04 | Citalopram | N06AX16 | Venlafaxine |
| N05BC01 | Meprobamate | N06AB05 | Paroxetine | N06AX17 | Milnacipran |
| N05BE01 | Buspirone | N06AB06 | Sertraline | N06AX18 | Reboxetine |
| N06AA01 | Desipramine | N06AB08 | Fluvoxamine | N06AX21 | Duloxetine |
| N06AA02 | Imipramine | N06AB10 | Escitalopram | N06AX22 | Agomelatine |
| N06AA04 | Clomipramine | N06AF01 | Isocarboxazid | N06AX26 | Vortioxetine |
| N06AA06 | Trimipramine | N06AF03 | Phenelzine | - | - |

Table S2 Diseases used for calculating Charlson comorbidity index

| **Disease** | **ICD-10 code** |
| --- | --- |
| **Myocardial infarction** | I21, I22, I252 |
| **Congestive heart failure** | I110, I130, I132, I50 |
| **Peripheral vascular disease** | I70, I71, I731, I738, I739, I771, I790, I792, K551, K558, K559, R02, Z958, Z959 |
| **Cerebrovascular disease** | I60-I69, G45, G46 |
| **Dementia** | F00, F01, F02, F03, F051, G30, G311 |
| **Chronic pulmonary disease** | J40-J47, J60, J61, J62, J63, J64, J65, J66, J67, J684, J70, J841, J920, J961, J982 |
| **Connective tissue disease** | M05, M06, M30, M315, M32, M33, M34, M351, M353, M360 |
| **Ulcer disease** | K25, K26, K27, K28 |
| **Mild liver disease** | B18, K700, K701, K702, K703, K709, K713, K714, K715, K717, K73, K74, K760, K762, K763, K764, K768, K769, |
| **Diabetes mellitus** | E100, E101, E106, E108, E109, E110, E111, E116, E118, E119, E120, E121, E126, E128, E129, E130, E131, E136, E138, E139, E140, E141, E146, E148, E149, |
| **Hemiplegia** | G041, G114, G801, G802, G81, G82, G839, G830, G831, G832, G833, G834 |
| **Moderate/severe renal disease** | I120, I131, N032, N033, N034, N035, N036, N037, N052, N053, N054, N055, N056, N057, N18, N19, N250, Z940, Z992 |
| **Diabetes mellitus with chronic complications** | E102, E103, E104, E105, E107, E112-E115, E122-E125, E132- E135, E142- E145, E117, E127, E137, E147 |
| **Any tumor** | C00-C14, C15-C26, C30-C34, C37-C41, C43, C45-C49, C50-C50, C51-C58, C60-C63, C64-C68, C69-C72, C73-C76, C97 |
| **Leukemia** | C91, C92, C93, C94, C95 |
| **Lymphoma** | C81, C82, C83, C84, C85, C88, C90, C96 |
| **Moderate/severe liver disease** | I85, K704, K72, K766 |
| **Metastatic solid tumor** | C77, C78, C79, C80 |
| **AIDS** | B20, B21, B22, B23, B24 |

Supplementary Figures


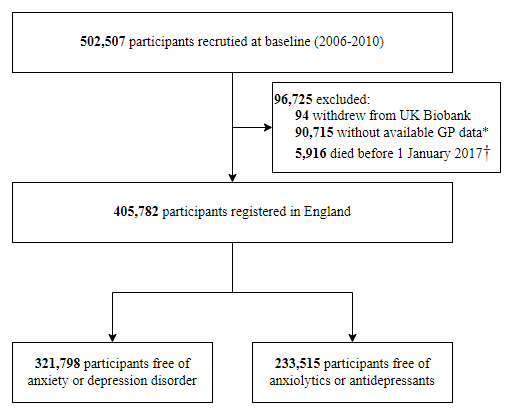


Fig. S1 Flow chart of the pre-pandemic reference population

* The primary care data were only available for participants registered in England and registered at a practice using TPP or EMIS as their data system supplier.

† Considering aging of the population, we used three preceding years as the pre-pandemic period (i.e., from 1 January 2017 to 31 December 2019).

**
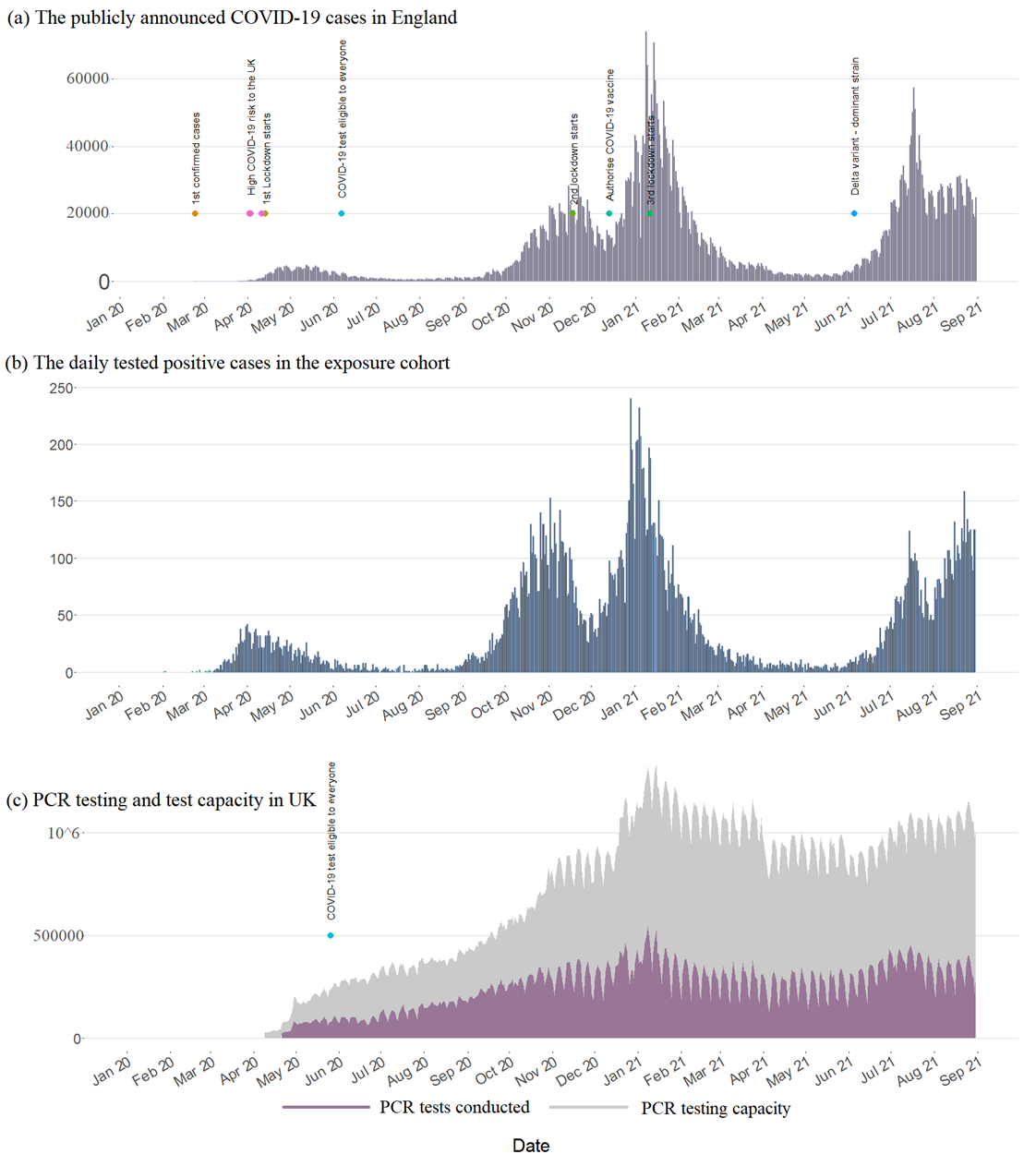
**

Fig. S2 The publicly announced COVID-19 cases in England and the PCR testing capacity in UK

Note, we obtained the publicly announced COVID-19 cases in England and PCR testing capacity from the official UK government website for data and insights on COVID-19 (<https://coronavirus.data.gov.uk/details/cases>).


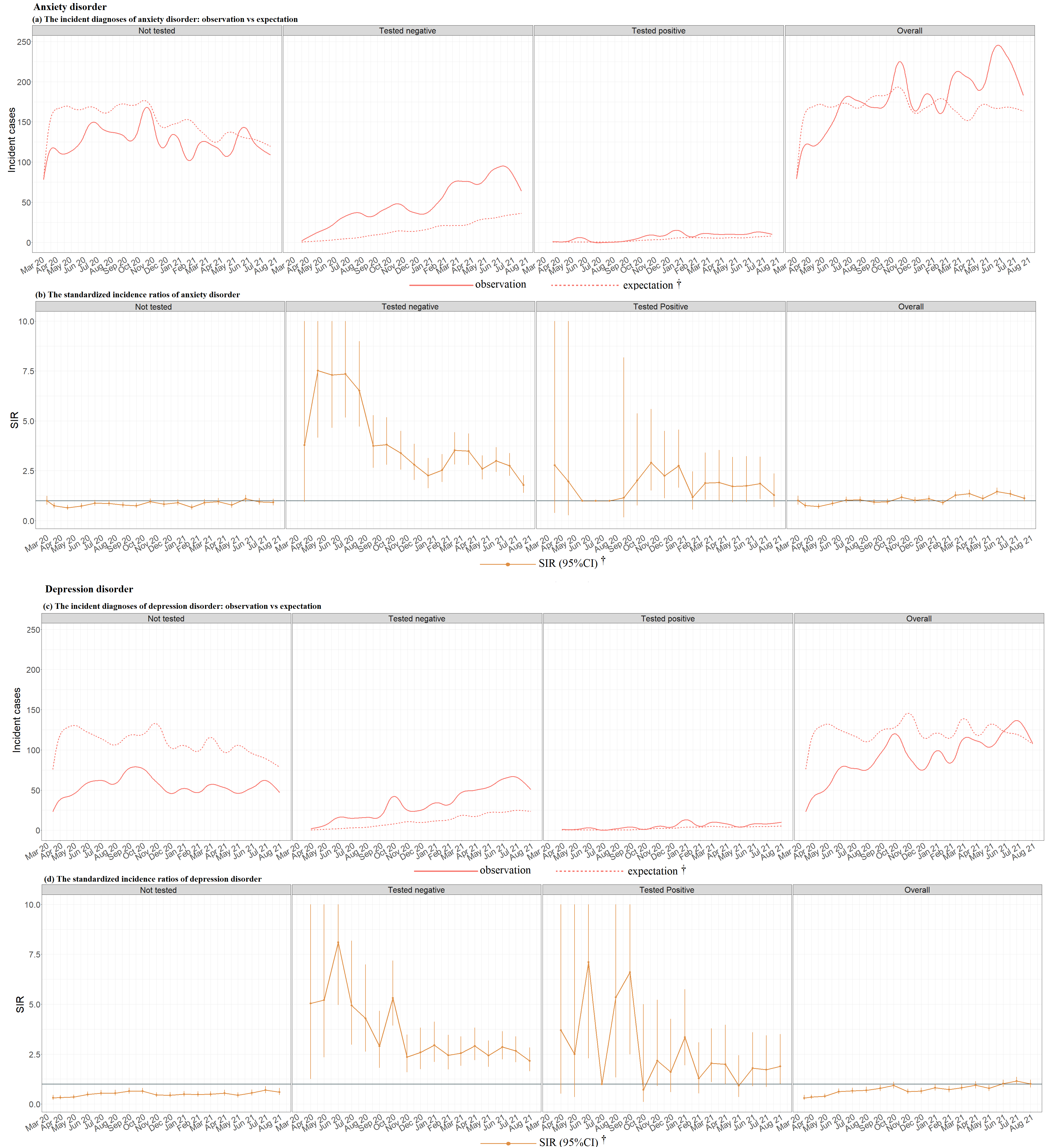


Fig. S3 Period specific incidence of anxiety and depression disorder: in subgroup analysis

* The expectation was calculated by multiplying the number of persons by the average month-specific, sex-specific, and age-specific (1-year strata) incidence rate derived from pre-pandemic period (i.e., from 1 January 2017 to 31 December 2019).

† SIR, standardized incidence ratio. SIR was calculated by comparing the number of observed incident cases with the expectation.


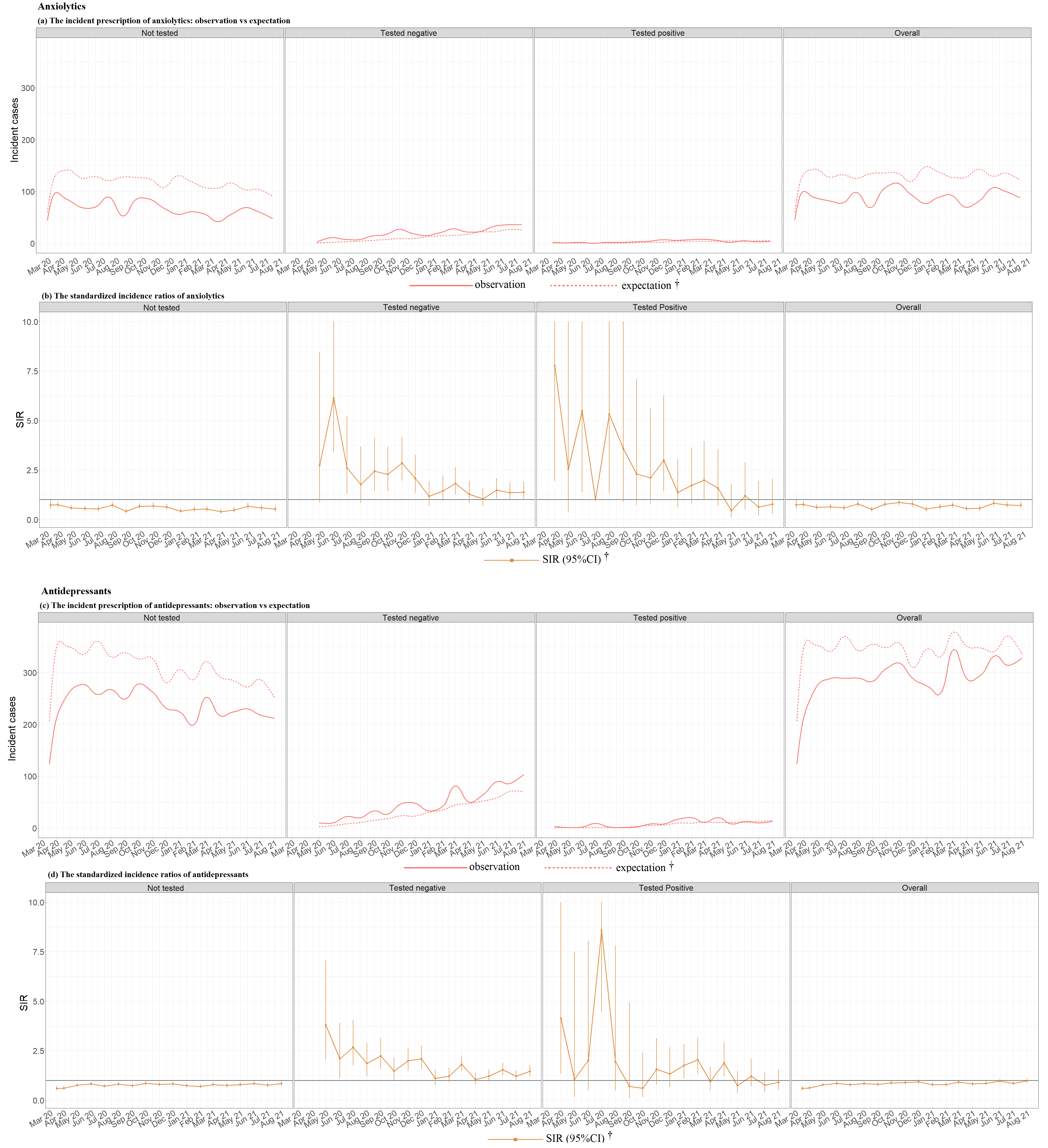


Fig. S4 Period specific incidence of anxiolytics and antidepressants prescription: in subgroup analysis

* The expectation was calculated by multiplying the number of persons by the average month-specific, sex-specific, and age-specific (1-year strata) incidence rate derived from pre-pandemic period (i.e., from 1 January 2017 to 31 December 2019).

† SIR, standardized incidence ratio. SIR was calculated by comparing the number of observed incident cases with the expectation.


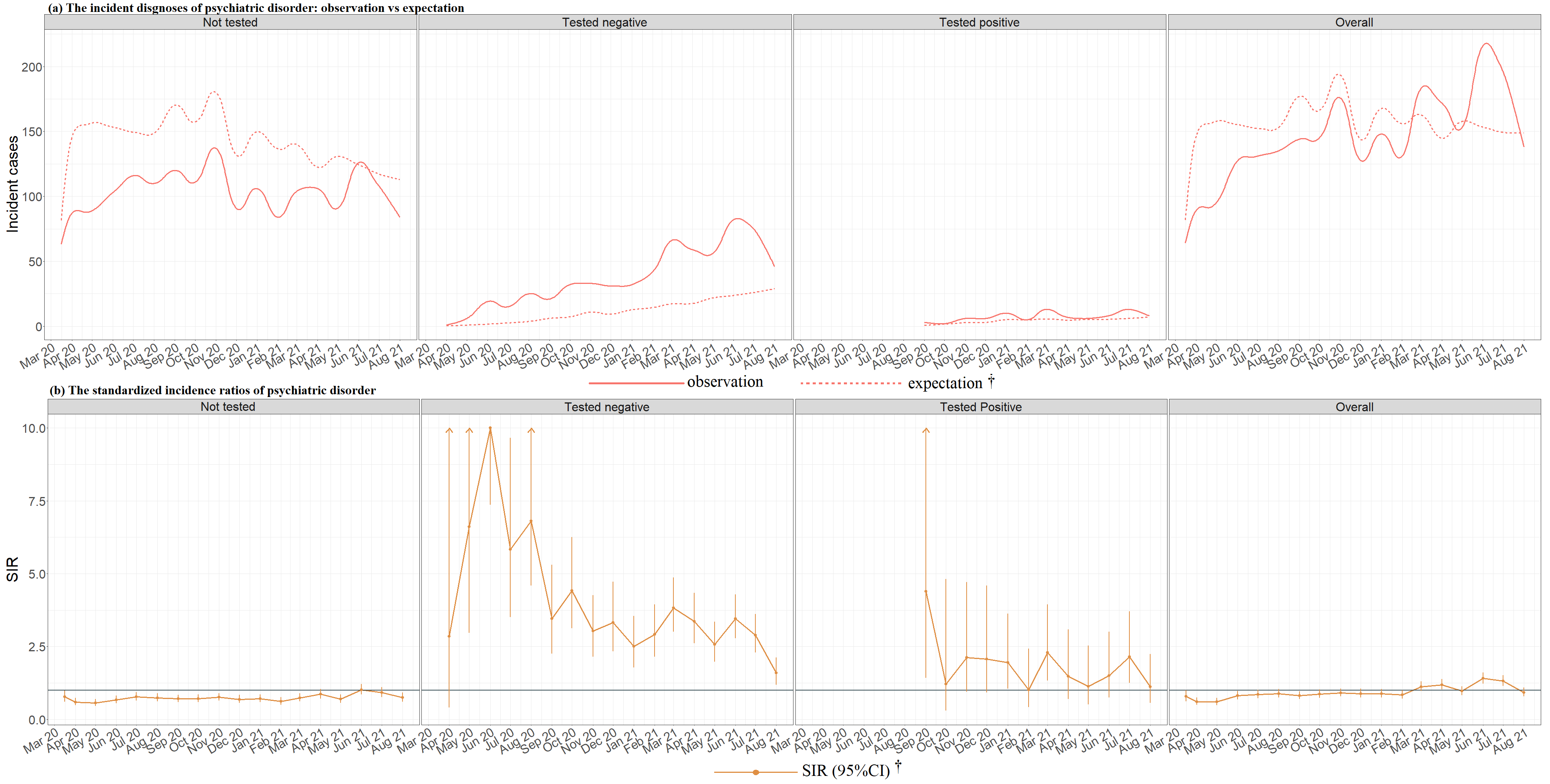


Fig. S5 Period specific incidence of anxiety and depression disorder: excluding participants with any severe somatic diseases (Charlson comorbidity index ≥1)

***** The expectation was calculated by multiplying the number of persons by the average month-specific, sex-specific, and age-specific (1-year strata) incidence rate derived from pre-pandemic period (i.e., from 1 January 2017 to 31 December 2019).

† SIR, standardized incidence ratio. SIR was calculated by comparing the number of observed incident cases with the expectatio.


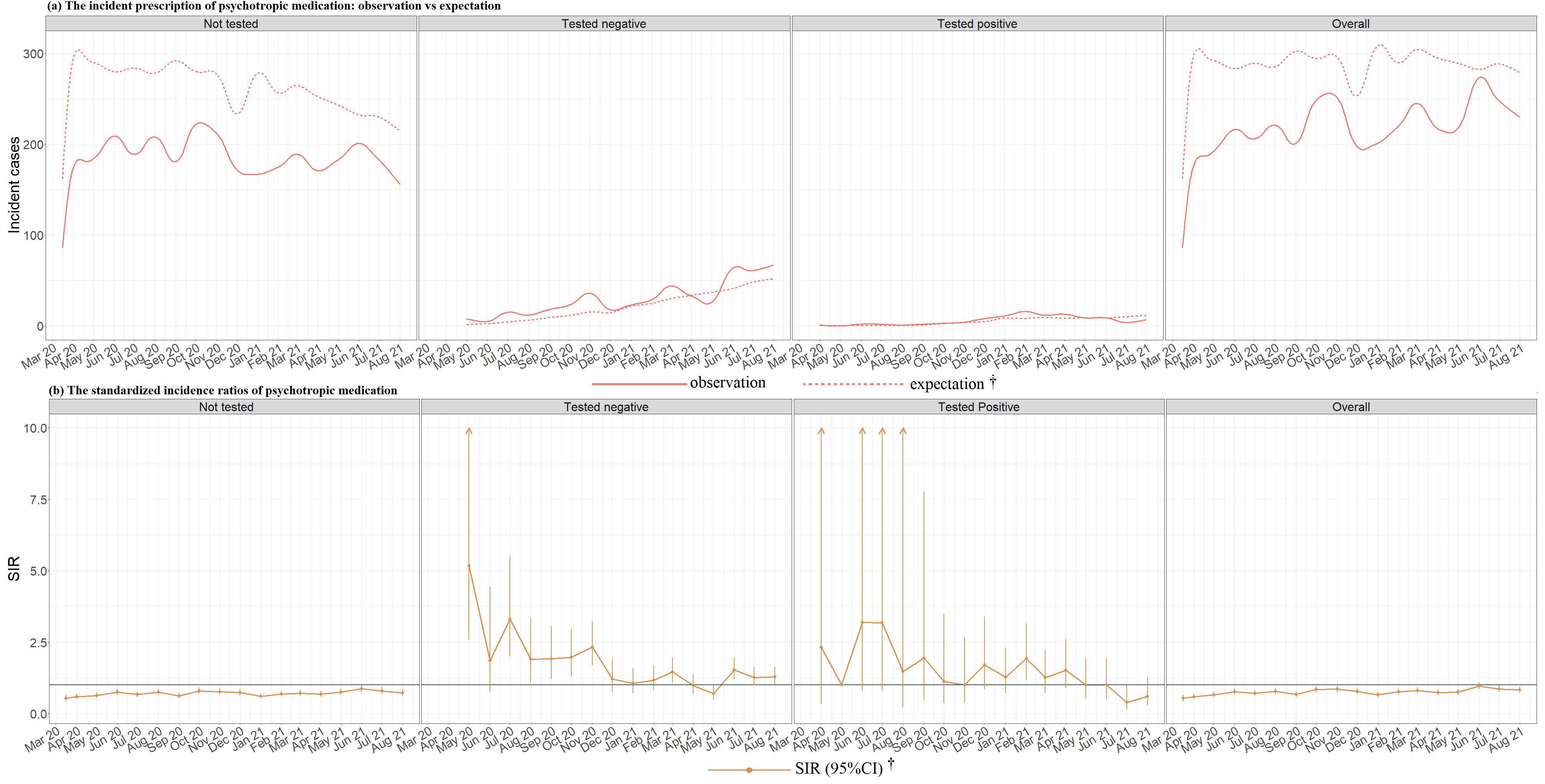


Fig. S6 Period specific incidence of anxiolytics and antidepressants prescription: excluding participants with any severe somatic diseases (Charlson comorbidity index ≥1)

***** The expectation was calculated by multiplying the number of persons by the average month-specific, sex-specific, and age-specific (1-year strata) incidence rate derived from pre-pandemic period (i.e., from 1 January 2017 to 31 December 2019).

† SIR, standardized incidence ratio. SIR was calculated by comparing the number of observed incident cases with the expectation.


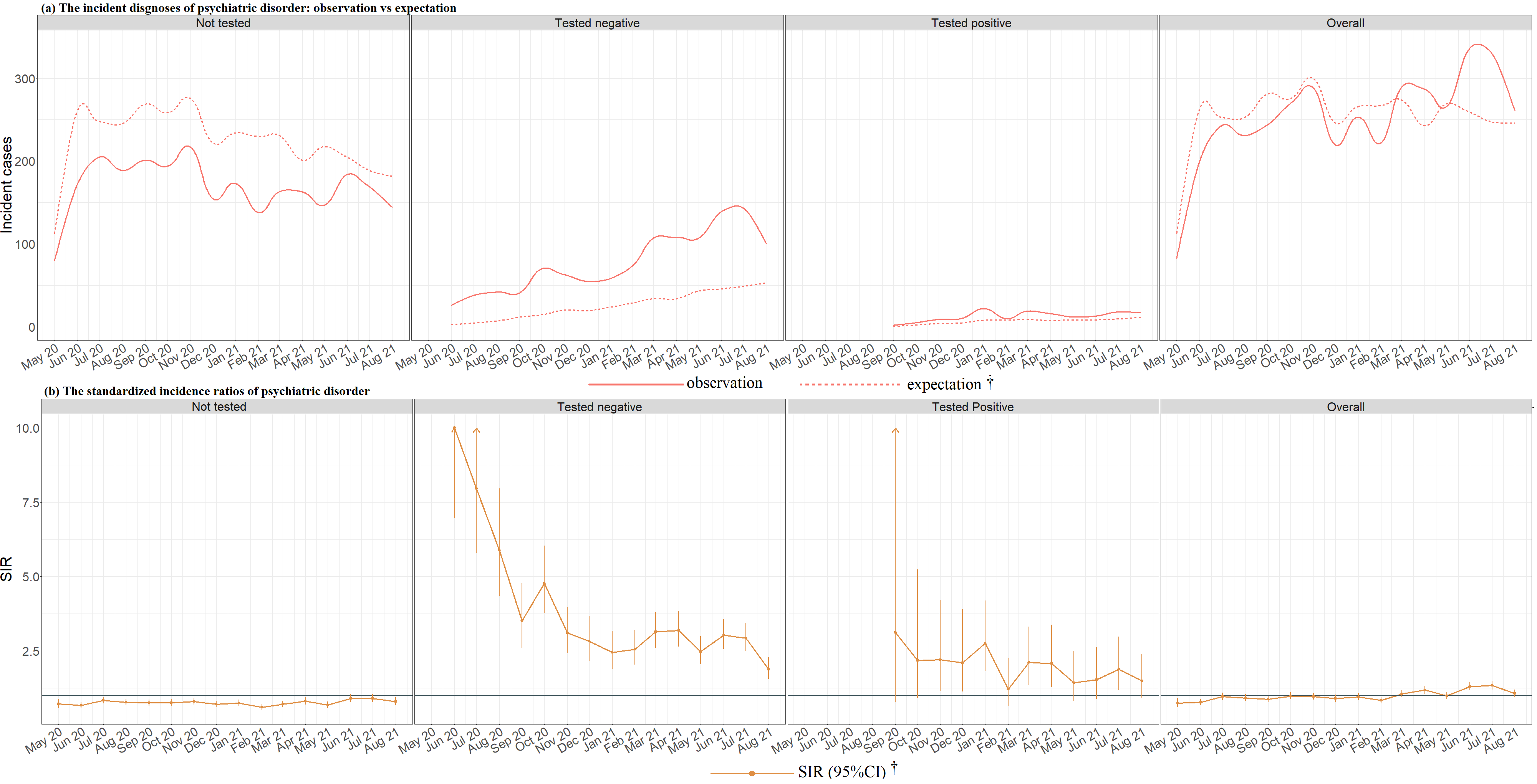


Fig. S7 Period specific incidence of anxiety and depression disorder: starting the pandemic period on 18 May 2020 (i.e, when COVID-19 test eligible to everyone with symptoms in the UK)

***** The expectation was calculated by multiplying the number of persons by the average month-specific, sex-specific, and age-specific (1-year strata) incidence rate derived from pre-pandemic period (i.e., from 1 January 2017 to 31 December 2019).

† SIR, standardized incidence ratio. SIR was calculated by comparing the number of observed incident cases with the expectation.


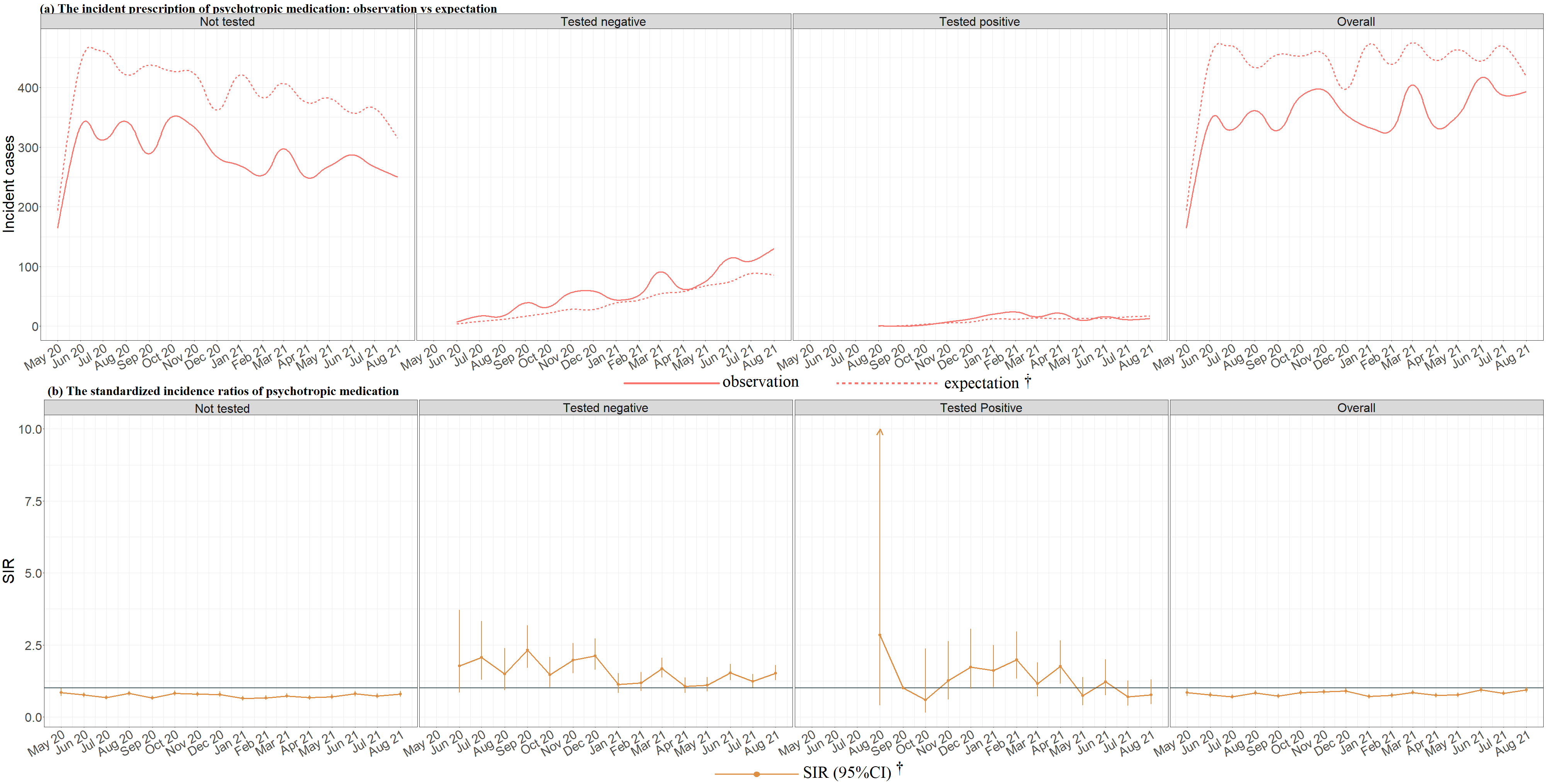


Fig. S8 Period specific incidence of anxiolytics and antidepressants prescription: starting the pandemic period on 18 May 2020 (i.e, when COVID-19 test eligible to everyone with symptoms in the UK)

***** The expectation was calculated by multiplying the number of persons by the average month-specific, sex-specific, and age-specific (1-year strata) incidence rate derived from pre-pandemic period (i.e., from 1 January 2017 to 31 December 2019).

† SIR, standardized incidence ratio. SIR was calculated by comparing the number of observed incident cases with the expectation.
